# Development and validation of an HPV infection knowledge assessment scale among Aboriginal and Torres Strait Islander Peoples

**DOI:** 10.1016/j.jvacx.2023.100317

**Published:** 2023-05-24

**Authors:** Sneha Sethi, Pedro Henrique Ribeiro Santiago, Gustavo Hermes Soares, Xiangqun Ju, Annika Antonsson, Karen Canfell, Megan Smith, Gail Garvey, Joanne Hedges, Lisa Jamieson

**Affiliations:** a Australian Research Centre for Population Oral Health, Adelaide Dental School, University of Adelaide, Adelaide, Australia; b School of Public Health, Faculty of Health and Medical Sciences, University of Adelaide, Adelaide, Australia; c QIMR Berghofer Medical Research Institute, Brisbane, Australia; d The Daffodil Centre, The University of Sydney, A Joint Venture with Cancer Council NSW; e Menzies School of Health Research, Charles Darwin University, Darwin, Australia

**Keywords:** Aboriginal and Torres strait islander health, Network analysis, HPV infection, Health promotion

## Abstract

**Background:**

An increased incidence of Human Papillomavirus (HPV) infection and its related cancers has been observed in recent years. Correct knowledge about HPV infection can lead to a significant decrease in transmission and a subsequent increase in vaccine uptake. Awareness and behavioural perception towards HPV infections are critical for improving HPV vaccination rates among Aboriginal and/or Torres Strait Islander Peoples. However, to the best of our knowledge, there has been no instrument designed to measure knowledge about HPV infection that is culturally appropriate and validated among Aboriginal and/or Torres Strait Islander People.

**Aim:**

To address this research gap, this paper aims to examine the psychometric properties of the HPV Knowledge Tool (HPV-KT) in an Indigenous population sample from South Australia.

**Methodology:**

Data from 747 Indigenous Australian Adults who participated in the 12-month follow-up of the HPV and Oropharyngeal Carcinoma in Indigenous Australians Study was utilised for this study. The psychometric properties examined included1) dimensionality and item redundancy; (2) network loadings; (3) model fit; (4) criterion validity; and (5) reliability. The network model was estimated using the Graphical Least Absolute Shrinkage and Selector Operator (GLASSO). Evaluation of the HPV-KT (10 items) dimensionality and item redundancy was conducted within the framework of Exploratory Graph Analysis (EGA). Reliability was evaluated with the McDonald’s Omega (ω) coefficient.

**Results:**

After the exclusion of two items, the HPV-KT exhibited good psychometric properties for Aboriginal and/or Torres Strait Islander Peoples. The two dimensions of “General HPV Knowledge” and “Commonness of HPV” were identified. The dimension of “Commonness of HPV” displayed poor reliability, so a sum score for this subscale is not recommended (i.e. the items can still be used individually) The network model of the 7-item HPV-KT was fitted in the validation sample and model fit was adequate (x2 (7) = 17.17, p < 0.016; CFI = 0.980; TLI = 0.94; RMSEA = 0.063, 90% CI = 0.025–0.010). Furthermore, the reliability of the “General HPV Knowledge” subscale (ω = 0.76, 95% CI: 0.72–0.79), while the reliability of the “Commonness of HPV” subscale (ω = 0.58, 95% CI0.58–0.88) was poor.

**Conclusion:**

The HPV-KT was adapted for an Aboriginal and/or Torres Strait Islander population and is readily available for future use in Australia. The addition of items assessing specifications of HPV infection, natural history and behaviour will improve the reliability and usability to assess the level of accurate knowledge about HPV infection. Future studies should investigate the possibility of developing new items for the dimension ‘Commonness of HPV’.

## Introduction

Human Papillomavirus (HPV) infections are recognised as among the most common sexually transmitted infections in the world, although generally asymptomatic [1], [2]. More than 200 subtypes of HPV [3] have been identified, of which 14 are recognised high-risk subtypes associated with cancers [4], with the rest low-risk types with HPV 6 and 11 being the most common [5], [6]. It has been reported that over 80% of sexually active individuals (men or women) will acquire an HPV infection before they turn 45 years of age, but these infections are often transient and are cleared without any clinical manifestations [7]. Persistent HPV infections are associated with a higher risk of initiating permanent changes in the cellular and tissue architecture, inciting a potentially malignant change [8], leading to an increase in HPV associated cancersworldwide [9], [10], [11].

HPV vaccines play a significant role in protecting against HPV infections, especially high-risk oncogenic types. The Food and Drug Administration (FDA) approved the safety and endorsed the provision of the vaccine against HPV infection in females (aged 9–26 years) in June 2006 and for males (aged 9–26 years) in October 2009 [12], [13]. In November 2020, the World Health Organization (WHO) launched the Global Strategy to Accelerate the Elimination of Cervical Cancer, the first of its kind in the eradication of cervical cancer. The WHO strategy endorses a “90–70–90” approach where 90% of the girls are vaccinated by the age of 15 years, 70% of women have access to high-performance screening tests for cervical cancer, and 90% of women with cervical cancer have adequate access to quality treatment by 2030 [14]. In 2007, Australia became one of the first countries to adopt and implement a National HPV Vaccination Programme, which is an ongoing school-based vaccination program for girls and boys [15], resulting in the lowest rates of cervical cancer worldwide [16]. However, regional disparities in HPV vaccination persist at both local and global levels [17], [18]. Limited knowledge and information about HPV infection have been recognised as one of the primary reasons for the low acceptance and uptake rates of HPV vaccine [14], [17], [19], [20].

A higher prevalence of HPV infections amongst Indigenous populations globally compared to the general population has been reported [21]. The increased persistence of HPV infection among Indigenous Peoples (Australia and Canada) can be attributed to a variety of factors including insufficient knowledge and lower screening uptakes [22], [23], [24], [25], [26], [27], [28]. A similar trend has been observed in Australia, where, despite the success of the Australian National HPV programme, Aboriginal and Torres Strait Islander Peoples continue to bear a large burden of HPV infection-associated cancer incidence, prevalence and mortality compared to other Australian people [23], [24], [25], [26], [27], [29], [30], [31]. United Nations Indigenous Peoples’ Partnership defines “Indigenous Peoples” as ‘people with a historical continuity with pre-invasion and pre-colonial societies that developed on their territories, and who consider themselves distinct from other sectors of the societies now prevailing on those territories’. At a global level, Indigenous Peoples embody 370 million people across 70 countries, and are known to experience health and social disadvantages that are unique compared to other minoritized population groups [32], [33]. The disadvantages experienced by Indigenous populations include loss of lands, identity, languages and control over life as part of a continuing intergenerational trauma that started with colonisation. Indigenous Peoples are victims of sustained racialisation and discernment with policies centred on cultural assimilation and personal annihilation [34], [35]. Indigenous Peoples are also over-represented among the poor and disadvantaged, especially in developed countries [36]. Nonetheless, Indigenous Peoples have demonstrated enormous resilience and have thrived in the contemporary world and Australia despite the history of colonisation and intergenerational trauma [37].

Awareness and behavioural perception towards HPV infections are critical for improving HPV vaccination rates among Aboriginal and Torres Strait Islander Peoples [38]. Hendry et al. (2013) reported that inaccurate perceptions of risk could impact HPV vaccination uptake, whereas precise HPV information could potentially increase protective behaviours, including safe sexual practices [39]. Successful interventions for HPV infection-associated cancer prevention are contingent on the knowledge and cognizance of HPV infection and various preventive strategies. To understand the current levels of HPV infection knowledge amongst Aboriginal and Torres Strait Islander populations, it is necessary first to develop (or adapt) and validate an instrument to measure HPV knowledge that is culturally appropriate for Aboriginal and Torres Strait Islander Peoples.

The development and validation of culturally-appropriate HPV knowledge instruments for Aboriginal and Torres Strait Islander Peoples are required since many Western-developed instruments can be culturally biased and, consequently, inadequate [40]. Other Western-developed instruments have been shown to be appropriate for Aboriginal and Torres Strait Islander Peoples after cultural adaptations [41], [42]. To establish that a developed or adapted instrument is correctly capturing HPV knowledge among Aboriginal and Torres Strait Islander populations, it is imperative to ensure its validity (whether the instrument measures what is supposed to measure) and reliability (whether the instrument is measuring what it is supposed to measure precisely without being strongly influenced by measurement error) [43]. While previous studies have developed and validated instruments measuring HPV knowledge among Western populations, such as in Italy and Canada [44], [45], [46], [47], [48], there have been no instruments validated specifically for Aboriginal and Torres Strait Islander Peoples. One of the instruments developed and validated for Western populations is the HPV Knowledge Tool (HPV-KT) [49]. The HPV-KT is a 29-item instrument that measures the domains of “general HPV knowledge”, “HPV testing knowledge” and HPV vaccination knowledge” [50]. Whilst there are no culturally-appropriate instruments validated to measure HPV knowledge among Aboriginal and Torres Strait Islander populations, the levels of HPV knowledge among Aboriginal and Torres Strait Islander Peoples is unclear. Given this research gap, the current study will investigate the psychometric properties of the HPV-KT in an Aboriginal and Torres Strait Islander population [49].

In the current study, to investigate the psychometric properties of the HPV-KT in an Aboriginal and Torres Strait Islander population, we employed a novel psychometric methodology, namely network psychometrics [50], [51]. Network psychometrics conceptualises that social, psychological, and biological components establish mutually reinforcing causal associations, while the use of network statistical models can provide a system-level description of how these social, psychological, and biological components are (conditionally) associated [52], [53]. A network model is visually depicted as nodes and edges, in which nodes represent items (measuring social, psychological and biological components) and edges represent conditional associations [53]. Furthermore, Christensen, Golino [51] discussed how instrument validity (e.g. dimensionality, item fit, model fit) can be examined entirely within the network perspective (without the need to rely on latent variables and their problematic assumptions [54]).

The current study aimed to employ network psychometrics to examine the psychometric properties of the HPV-KT in an Aboriginal and Torres Strait Islander population. The psychometric properties investigated will be: (1) dimensionality and item redundancy; (2) network loadings; (3) model fit; (4) criterion validity; and (5) reliability.

## Methods

### Data collection and study design

Data were collected from a prospective longitudinal cohort study that aims to estimate the prevalence, incidence, clearance and persistence of oral HPV infection and identify risk factors associated with oropharyngeal carcinoma among Indigenous Australians [51]. The study was conducted in partnership with chief Indigenous stakeholder groups, and governed by an Indigenous Reference Group. The study received ethical approval from the University of Adelaide Human Research Ethics Committee (H-2016–246) and the Aboriginal Health Council of South Australia (04–17–729) [51]. All participants provided written informed consent.

Inclusion criteria included being aged 18 + years and identifying as being Aboriginal and/or Torres Strait Islander. Baseline data was collected from February 2018 to January 2019 (N = 1011 participants) with 12-month follow-up data collected from February 2019 to January 2020 (N = 747 participants) and 24-month follow-up data collected from February 2020 to January 2021 (N = 803 participants). All components of data collection, including sensitive sexual behaviour questions, were pilot-tested and tailored for cultural sensitivity. Pilot testing was performed amongst five Indigenous members of the Indigenous Oral Health Unit Advisory group. Information on sociodemographic factors, sexual behaviours, and health-related behaviours were ascertained by self-report questionnaire, while the study's Senior Indigenous Research Officer (J. Hedges) assisted participants where required (e.g. if respondents had questions about item wording). For purposes of this study, data from the 12-month follow-up was used. Further information on the HPV and Oropharyngeal Carcinoma in Indigenous Australians Study can be found elsewhere [31], [51].

### Primary measure (HPV knowledge Tool)

The HPV Knowledge Tool (HPV-KT) is a 29-item instrument originally developed and validated by Waller, Ostini [49] in a sample of Australian, American and British adults. The HPV-KT included 29 items measuring the three dimensions of: general HPV knowledge (16 items); HPV testing knowledge (6 items); and HPV vaccination knowledge (7 items). Overall, the HPV-KT aims to capture the individual level of understanding of HPV infection, the mode of transmission and the associated cancer risk. The items are rated on a dichotomous scale (“True” and “False”) and a “Don’t know” option is also displayed. For example, the question “HPV is very rare” can be answered as “True”, “False”, or “Don’t know”. Each question has a correct answer (e.g. the question “HPV is very rare” should be rated as “False”). The total score is obtained by summing all correct responses, with higher scores indicating stronger knowledge about HPV [49]. After its development, McBride, Marlow [50] implemented a short-form version containing 10 items in the National Health Service Cervical Screening Programme (NHSCSP) in the UK. The short-form 10-item version includes only items from the “general HPV knowledge” domain of the original 29-item HPV-KT instrument. To the best of our knowledge, the psychometric properties of this short-form 10-item version have not been evaluated in any population.

In the HPV study, a modified HPV-KT short-form 10-item version was implemented. Based on recommendations for cultural adaptation of instruments [52], prior to implementation of the HPV-KT in the HPV and Oropharyngeal Carcinoma in Indigenous Australians Study (henceforth referred to as the “HPV study”), the HPV-KT was adapted for Aboriginal and Torres Strait Islander adults based on recommendations from an Indigenous Reference Group. The Indigenous Reference Group investigated the instrument content and face validity, acceptability, and item comprehension, among other psychometric properties. The Indigenous Reference group endorsed the usage of the 10-item HPV-KT in its current form and only suggested modifications to one item. Considering that the HPV study included both men and women (the NHSCSP, for instance, included only women [50]), the Indigenous Reference Group suggested that the item “HPV can cause cervical cancer” should be reworded to “HPV can cause cancer in men” to check whether participants understood that HPV can also cause cancer in men (i.e. a common misconception is that “only ‘promiscuous women’ get HPV” [53]). As such, we followed the recommendations from the Indigenous Reference Group and reworded this item.

In the current study, correct responses to each item were coded as 1, while incorrect and “Don’t know” responses were coded as 0. The reason that “Don’t know” responses were coded as 0 was because evidence shows that “not knowing” information about HPV (or others diseases) is as detrimental as knowing incorrect information in terms of relevance for prevention strategies, attitudes towards vaccination, etc. For example, a common misbelief supporting vaccine hesitancy is that “not knowing something is false is proof it is true” [54]. The three items conveying false information on HPV (Items 1, 2 and 6) were reverse coded (“True” and “Don’t-know” were coded as the incorrect response and “False” as the correct response) to ensure uniform coding structure across all the items (i.e. all correct responses were coded as 1, and all incorrect responses were coded as 0). All item labels are displayed in Table 2.

### Secondary measures (sociodemographic characteristics and perceived knowledge of HPV)

Sociodemographic characteristics included sex, age and areas in which participants lived (rural, remote or metropolitan area). The Rural, Remote and Metropolitan Area (RRMA) classification divides Australia into rural, remote, and metropolitan areas and the area was identified based on the study participants’ postcodes. To investigate criterion validity, the participants’ perceived knowledge of HPV was evaluated through the question “Do you think your knowledge about HPV is…” rated based on six response categories (0= “Never heard of HPV”; 1=“Very poor”; 2=“Poor”; 3=“Fair”; 4=“Good”, and 5=“Very good”). Higher scores on the perceived knowledge of HPV item indicate a higher perceived knowledge of HPV.

### Statistical analysis

Initially, the distribution of responses to the HPV-KT tool was examined through mean, standard deviation, kurtosis, skewness, and proportion of correct and incorrect responses. Considering that the proportion of missing values on individual items was smaller than 3% across all items, we followed guidelines that multiple imputation on the response sample (n = 747) was not required and all analyses were conducted on the complete case sample (n = 721). We then proceeded to evaluate: (1) dimensionality and item redundancy; (2) network loadings; (3) model fit; (4) criterion validity; and (5) reliability. The statistical analysis was conducted with R software [55] and the R packages EGAnet [56] and psychometrics [57].

The network model was estimated using the Graphical Least Absolute Shrinkage and Selector Operator (GLASSO) and the tuning parameter γ was chosen based on the minimization of the Extended Bayesian Information Criterion (EBIC). Given the ordinal nature of the HPV-KT items, the polychoric correlation matrix was used as input [58]. Following best practices in reporting, we report the polychoric correlation matrix of the (final) network model in Supplementary Table 1 [59]. We also calculated two centrality indices, *strength centrality* and *expected influence*, as standardized z-scores [60]. Items were graphically represented as nodes and partial correlations between pairs of items were illustrated as edges connecting the respective nodes. Partial correlations provide information on the strength and direction of the conditional associations between two items (nodes) whilst controlling for all the other items included in the network. Positive partial correlations were plotted as blue edges, and negative partial correlations were plotted as red edges. The strength of the partial correlation between items is represented by the edge width.

**Table 1:** Socio-Demographic characteristics of participants.

Socio-demographic variables		
	Response sample (n = 747)	Complete case sample (n = 721)
Age	18–79 (41.93 ± 12.17)	18–79 (40.84 ± 14.63)
Sex *Male* *Female*	224 (31.0)497 (68.9)	224 (31.0)497 (68.9)
Location *Rural* *Metro*	443 (61.3)278 (38.3)	443 (61.3)278 (38.3)
Education *Till High school* *Trade or University*	496 (66.4)251 (33.6)	480 (66.5)241 (33.4)
Income *Job or other* *Healthcare*	233 (31.2)514 (68.8)	233 (31.2)514 (68.8)
Health Care card *Yes* *No*	547 (73.3)200 (26.7)	531 (73.6)190 (26.3)
Smoking Status *Non-smoker* *Current Smoker* *Former smoker*	211 (28.4)384 (51.4)152 (20.1)	201 (27.8)374 (51.8)146 (20.2)
Alcohol consumption *Daily* *Weekly* *Monthly* *Never*	49 (6.6) 175 (23.4)259 (34.7)264 (35.3)	47 (6.5) 170 (23.5)250 (34.6)260 (36.0)
Ever diagnosed with HPV*? *Yes* *No or don’t know*	23 (3.1)724 (96.9)	17 (2.4)704 (97.6)
HPV* Vaccination *Received* *Not received or don’t know*	62 (8.3)685 (91.7)	56 (7.7)665 (92.2)
Ever heard of HPV* before today? *Yes* *No*	422 (56.4)325 (43.5)	406 (56.3)315 (43.7)
Any unanswered questions about HPV*? *Yes* *No*	219 (29.3)528 (70.6)	200 (27.7)521 (72.3)
Ever offered a vaccine for HPV*? *Yes* *No or not sure*	558 (74.6)189 (25.3)	543 (75.4)178 (24.6)
Self-rated general health *Excellent* *Very good* *Good* *Fair or poor*	80 (10.7)185 (24.7)297 (39.7)185 (24.8)	77 (10.6)176 (24.4)289 (40.0)179 (24.8)
Self-rated oral health -*Excellent* *-Very good* *-Good* *- Fair or poor*	74 (9.9)129 (17.4)266 (35.6)278 (37.1)	72 (9.9)126 (17.4)255 (35.3)268 (37.1)

**Table 2:** Distribution of participants’ responses to HPV-KT items (N = 747).

Item	Label	Description	Mean	SD	Skewness	Kurtosis	Correct response (%)
1	*rare*	*HPV is very rare*	0.22	0.41	1.32	−0.24	22.3
2	*signs*	*HPV always has visible signs or symptoms*	0.22	0.41	1.31	−0.26	22.1
3	*skin*	*HPV can be passed on by genital skin-to-skin contact*	0.32	0.46	0.75	−1.44	32.7
4	*types*	*There are many types of HPV*	0.34	0.47	0.63	−1.60	34.5
5	*sex*	*HPV can be passed on during sexual intercourse*	0.47	0.49	0.09	−1.99	47.7
6	*men*	*Men cannot get HPV*	0.47	0.49	0.08	−1.99	47.8
7	*treatment*	*HPV usually doesn’t need any treatment*	0.09	0.29	2.73	5.47	9.7
8	*lives*	*Most sexually active people will get HPV at some point in their lives*	0.31	0.46	0.81	−1.34	30.7
9	*know*	*A person could have HPV for many years without knowing it*	0.51	0.50	−0.05	−2.00	51.1
10	*cancer*	*HPV can cause cancer in men*	0.34	0.47	0.63	−1.59	34.6

The evaluation of the HPV-KT dimensionality and item redundancy was conducted within the framework of Exploratory Graph Analysis (EGA) [61]. Firstly, we evaluated item redundancy with the weighted topological overlap (wTO) statistic with adaptive alpha [62]. In case wTO indicates potential redundancy, the item content was examined to evaluate whether the items measured the same “construct” (i.e. a unique causal component in the network). If the items were truly redundant, one of the redundant items was removed. If the items were similar in content (due to measuring the same “construct”) but conceptually different enough to preclude item exclusion, the items were summed together generating a composite item (i.e. “super item”) [63].

The Walktrap algorithm was used to identify the network communities and investigate dimensionality [64]. (TEFI; 65]. The unidimensionality check was conducted with the Leading Eigenvalue algorithm [65]. In the visual representation of the network model, node colours represent items that belong to the same community. Once a community structure was identified, we proceeded to evaluate how stable was this structure to random sampling variation [66].

The stability of the communities identified in the HPV-KT network was evaluated using Bootstrap Exploratory Graph Analysis (bootEGA) [67]. This method repeatedly re-samples the original data to produce a sampling distribution of HPV-KT network to assess the stability of dimensions identified by the Walktrap algorithm. The number of bootstrap samples was set to 1,000. Item stability (with values ranging from 0% to 100%) provides information on the proportion of times that an item clustered (in the bootstrap samples) with the community identified in the original sample [67]. Dimensions were considered structurally consistent (and items considered stable) when exact replicates occurred across 75% or more of the bootstrap samples [68].

The fourth step was the evaluation of network loadings. Network loadings can be small (from 0.0 to 0.15), moderate (0.16 to 0.25), or large (0.26 to 0.35). Items that did not load substantively on any network community (<0.16 across all communities) or had substantive cross-loadings (>0.15 in more than one community) were considered for exclusion (70].

To avoid overfitting due to capitalisation on sampling variation, we used a data-splitting procedure to divide the sample into development and validation samples [70]. The complete case sample was randomly split into a test sample (n = 360) and a validation sample (n = 371). The exploratory steps used to define the model and potentially remove items (dimensionality and item redundancy and evaluation of network loadings) were conducted on the test sample, while the confirmatory steps used to evaluate model fit, criterion validity and reliability were conducted on the validation sample. To evaluate model fit, we examined whether the estimated network model (in the test sample) adequately explains the observed polychoric correlations among variables (in the validation sample) [71]. Model fit was evaluated according to following fit indices: the χ2 statistic, Comparative Fit Index (CFI), and the Root Mean Square Error of Approximation (RMSEA). We followed conventional guidelines that RMSEA values ≤ 0.05, and CFI values > 0.96 were considered indicative that the model provides a good fit to the data, RMSEA values between 0.05 and 0.10 were considered as adequate fit, whereas RMSEA values above 0.10 were deemed unacceptable.

To evaluate reliability, we employed McDonald’s Omega (ω) coefficient since it has several advantages over other more traditionally used coefficients, such as Cronbach’s Alpha (α) [72], [73], [74]. (76].Values of McDonald’s Omega above 0.70 are adequate for research purposes.

To evaluate criterion validity, we calculated the Spearman correlation coefficients between the network community scores [69] and perceived knowledge of HPV (“Do you think your knowledge about HPV is…”). It was expected that participants with higher scores on the HPV-KT (i.e. higher knowledge about HPV) would present higher scores on perceived knowledge of HPV. A nonparametric bootstrapping procedure was employed to calculate the corresponding 95% Confidence Intervals (95% CIs).

## Results

### Sample characteristics

The population sample included 747 adults aged 18–79 years (mean 41.93 ± 12.17 years; median 51 years). Participants were predominantly female (68.9%), living in non-metropolitan areas (61.3%), and without tertiary education (66.4%). Approximately 56.3% of participants reported having heard about HPV, whereas 72.3% reported having no additional questions about HPV. More than 20% of the participants reported having never been offered the vaccine against HPV. There were no substantive differences between the response sample and the complete case sample (Table 1).

The distribution of items’ responses to the HPV-KT tool is summarized in Table 2. Mean item scores ranged from 0.09 (Item 7) to 0.51 (Item 9). Item scores presented a right-skewed distribution (skewness ranged from −0.05 to 2.73).

### Dimensionality, item redundancy and network loadings (test sample)

**Fig. 1:**
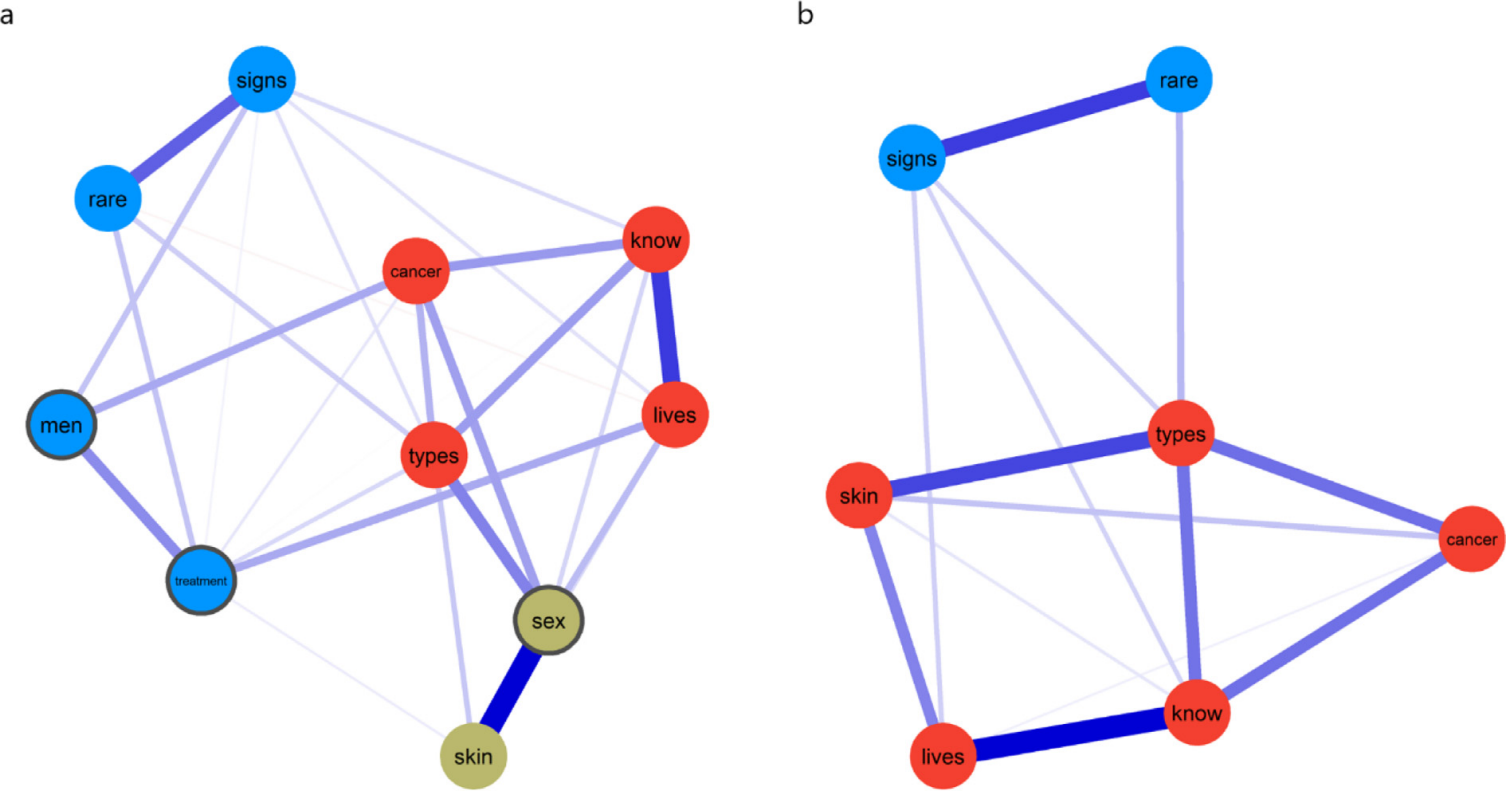
**Initial and final network models of the HPV-KT scale***Note. The initial model is displayed on panel a. The final model is displayed in panel b. Nodes represent items and edges represent partial correlations between items. Nodes with grey borders indicate items that were excluded during the development stage.*

*Ten-item network*: The 10 items HPV-KT scale formed a single network structure comprising three node communities (Fig. 1). The strongest edges in the network emerged between items 3 (*skin*) and 5 (*sex*), and items 8 (*lives*) and 9 (*know*). EGA detected a node community comprising only two items (3 and 5). One possible reason for a community comprised of only two items is item redundancy. Item stability (Fig. S1) and node centrality (Fig. S2) are displayed in Supplementary Material.

We proceeded to evaluate item redundancy. The strongest weighted topological overlap was 0.470 (p = 0.002), observed for items 3 (*skin*) and 5 (*sex*) and indicating potential redundancy. After examination of the item content, we decided to exclude Item 5 (*sex*) since Item 3 (*skin;* “HPV can be passed on by genital skin-to-skin contact”) also measures knowledge about sexual transmission of HPV and specifies that skin-to-skin contact is required for infection transmission (since transmission does not occur when male or female condoms are used). As such, Item 3 was more general while measuring the same knowledge about sexual transmission of HPV and it was retained.

*Nine-item network:* Following the exclusion of Item 5 (*sex*), the network model was re-estimated. The Walktrap algorithm identified three communities, providing evidence that the third community identified (when all ten items were considered) was spurious due to item redundancy. The first community consisted of items 1 (*rare*), 2 (*signs*), 6 *(men*) and 7 (*treatment*), whereas the second community comprised items 3 (*skin*), 4 (*types*), 8 (*lives*), 9 (*know*) and 10 (*cancer*). The network model (Fig. S3), item stability (Fig. S4), node centrality (Fig. S5) are displayed in the Supplementary Material. Adequate structural consistency was observed for the second community (92.1%), but not for the first community (71.4%). The analysis of item stability measures indicated that items 6 (men = 74.0%) and 7 (treatment = 72.0%) had low stability and were the source of the low structural consistency of the first community (Fig. S4). Furthermore, Item 7 also presented substantial cross-loading (Supplementary file). Given the low stability of these two nodes and the substantive cross-loadings of Item 7 (these items did not clearly belong to a unique network community), these items were removed and we proceeded to re-estimate the network.

**Fig. 2:**
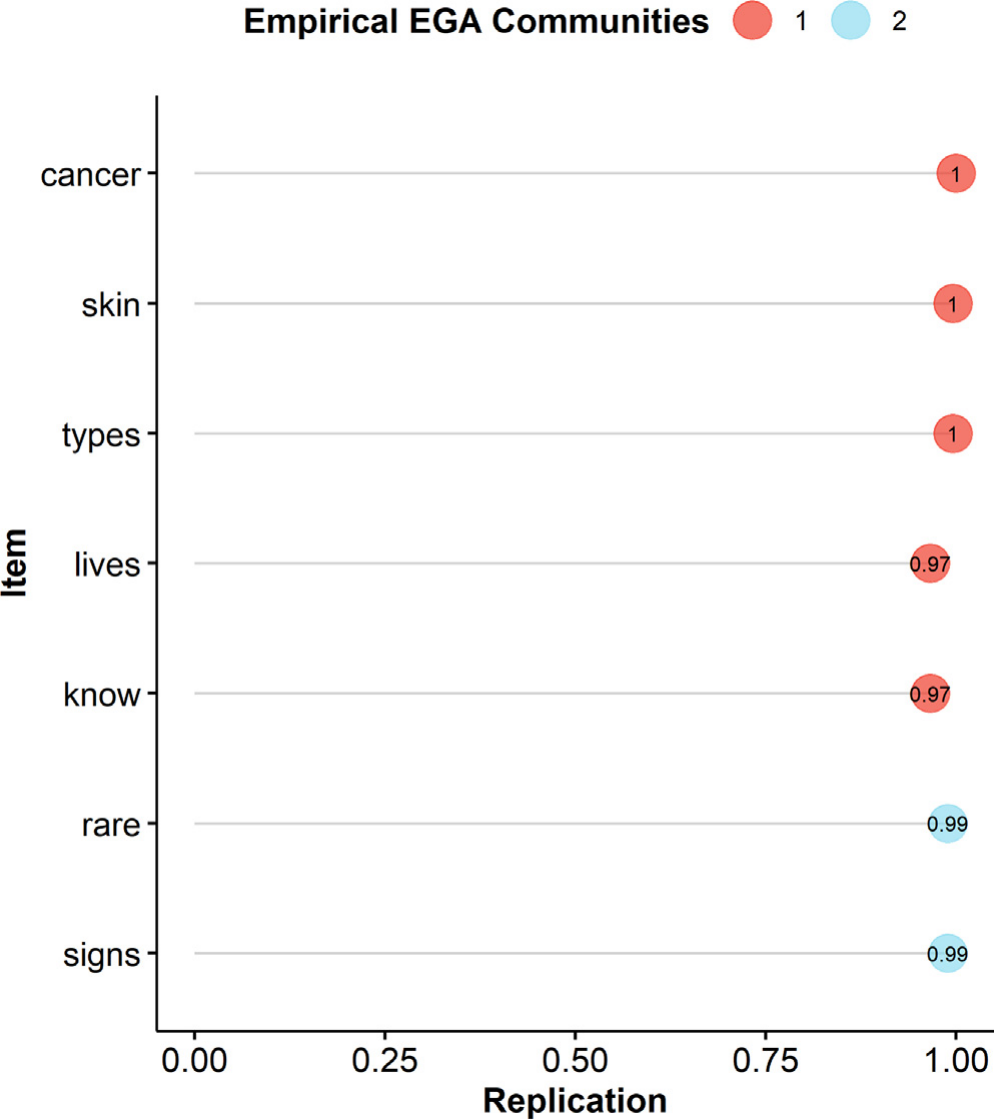
**Item stability of the final HPV-KT network model***Note. The y-axis indicates the items. The circles are coloured according to their Walktrap-identified community. The x-axis indicates the proportion of times the item clustered with the Walktrap-identified community across the bootstrap samples. The number inside the circle indicates the proportion of times the item clustered with the Walktrap-identified community for each individual item.*

*Seven-item network:* Following the exclusion of items 6 and 7, the HPV-KT network model was re-estimated. The Walktrap algorithm identified again two communities (Fig. 1). The structural consistency of both communities improved considerably (Community 1: 98.9%; Community 2: 96.2%). Optimal item stability ranging from 97.0% to 100.0% was obtained for all items (Fig. 2). The node centrality (Fig. S2) is displayed in the Supplementary Material. The two communities were interpreted as “General HPV Knowledge” (Community 1) and “Commonness of HPV” (Community 2).

### Model fit, reliability and criterion validity (validation sample)

The network model of the 7-item HPV-KT was fitted in the validation sample and model fit was adequate (x^2^ (7) = 17.17, p < 0.016; CFI = 0.980; TLI = 0.94; RMSEA = 0.063, 90% CI = 0.025–0.010). Furthermore, the reliability of the “General HPV Knowledge” subscale (ω = 0.76, 95% CI: 0.72–0.79), while the reliability of the “Commonness of HPV” subscale (ω = 0.58, 95% CI: 0.58–0.88) was poor. Finally, the Spearman correlation coefficients between HPV-KT network community scores and perceived knowledge of HPV scores for both “General HPV Knowledge” subscale (ρ: 0.42; 95% CI: 0.33–0.50) and “Commonness of HPV” subscale (ρ: 0.44; 95% CI: 0.35–0.53) were positive and substantive. As such, the associations observed between the HPV-KT network community scores and perceived knowledge of HPV scores were according to theoretical expectations, providing initial evidence for the HPV-KT criterion validity.

## Discussion

HPV infection is highly prevalent and has been increasingly associated with the incidence of several cancers, such as cervical cancer and oropharyngeal cancer. Knowledge about HPV infection can lead to a significant decrease in transmission and greater vaccine uptake. However, all instruments validated to measure knowledge about HPV were developed in Western countries and no instrument had yet been culturally validated for Aboriginal and Torres Strait Islander Peoples. The current study aimed to assess the psychometric properties of a 10-item tool, the HPV-KT, to measure the knowledge of HPV infection in Aboriginal and Torres Strait Islander adults from South Australia. After the removal of three items, the HPV-KT exhibited adequate psychometric properties. This study identified two dimensions, which assessed general knowledge about HPV infection and knowledge about the commonness of HPV infection among Aboriginal and Torres Strait Islander Peoples, respectively. The 7-item HPV-KT is readily available for use among Aboriginal and Torres Strait Islander populations and guidelines on instrument use are provided.

Psychometric validity is critical to assess the potential of an instrument and ascertain if it is accurately and reliably measuring what it claims to be measuring. Warner et al. [80] assessed the knowledge of HPV infections and vaccinations among a small sample of Indigenous Caribbean women using six single-item measures. Although an informative and significant step towards HPV infection education highlighting the importance of culturally appropriate instruments, the instruments used in the study were limited in accurately measuring knowledge of HPV.

Regarding the HPV-KT psychometric properties among Aboriginal and Torres Strait Islanders, after the exclusion of one redundant item and two items with low item stability and cross-loadings, the analysis indicated that the network of the 7-item HPV-KT comprised two communities (“General HPV Knowledge” and “Commonness of HPV”) and displayed good psychometric properties. Among the excluded items, it should be noted that Item 7 (“HPV usually doesn’t need any treatment”) measured knowledge about treatment. Item 7 was excluded due to it displaying low item stability and substantial cross-loadings between the two dimensions, so it was not clear which dimension (“General HPV Knowledge” or “Commonness of HPV”) this item was measuring. Furthermore, Item 7 was the only item that displayed strong floor effects since 90.3% of the respondents answered the item incorrectly. At this point, it is unclear whether most respondents believed that HPV usually doesn’t need any treatment or whether the item wording was confusing to respondents. It is important that future studies further investigate the psychometric properties of this item in other Aboriginal and Torres Strait Islander samples and also develop and investigate the validity of other items designed to measure knowledge about HPV treatment among Aboriginal and Torres Strait Islander peoples.

The dimension identified as “Commonness of HPV” consisted of two items that clustered together in the network. One item measured the knowledge regarding the rarity or commonness of carrying HPV infection, whereas the other item measured knowledge regarding the commonness of the appearance of signs and symptoms associated with an HPV infection. These were the only two items in the final 7-item version of the instrument that were reverse-coded (i.e. the correct response to both of these items was “False”) so the “Commonness of HPV” could potentially be a “method factor” due to the common item valence [75]. Future studies need to further investigate this issue, and could use recently developed methods such as Random-Intercept Exploratory Graph Analysis (riEGA) [76], to examine whether the “Commonness of HPV” community is a theoretically substantive dimension of knowledge about HPV or a “method factor” due common item valence. The second dimension identified was the “General HPV Knowledge” dimension, most of the items (five) belonged to this dimension.

While the reliability of the “General HPV Knowledge” subscale was good, the reliability of the “Commonness of HPV” subscale was poor. One potential reason for the poor reliability was that the “Commonness of HPV” subscale included only two items. As such, while a subscale score can be computed for the “General HPV Knowledge” subscale, we advise *against* computing a subscale score for the “Commonness of HPV” subscale. Future studies should consider including new items in the “Commonness of HPV” subscale. Items that could potentially strengthen the reliability of the “Commonness of HPV” subscale could be questions related to sexual transmission, oral-sexual behaviours, the common appearance of lesions, threat from persistent HPV infections and probablity of cancer. Nonetheless, this does not preclude the use of the 7-item HPV-KT among Aboriginal and Torres Strait Islander adults. For instance, the *item score* of the two items (*rare* and *signs*) can be used independently in future research (i.e. measuring whether respondents understand how rare HPV is and if it has visible signs or symptoms) without the need to necessarily sum them into a subscale score [63].

In the current study, despite 72.2% of the cohort reporting to have sufficient knowledge about HPV and had no unanswered questions about it, the prevalence of oral HPV infection was high [31]. These figures might be indicative of exposure to limited and superficial information about HPV in the current sample. Fuenmayor et al, also found a vast gap of knowledge and awareness regarding sexually transmitted infections, contraception methods and HPV in particular [77] amongst Indigenous women from Maniapure, Bolivar State.

HPV vaccines and infections are a currently upcoming topic and only recently has there been much efforts by healthcare services and workers to provide information and awareness regarding the same. Our current study recruited only adults above the age of 18 years and the views as well as awareness of HPV infections and transmissions from the younger generations was completely missed. Although it was beyond the scope of the current study, future research with a focus on validating a tool for HPV infection, transmission and vaccine awareness amongst the younger generations is highly recommended.

To the best of our knowledge, this is the only study that has validated an instrument using recent psychometric methods (network analysis), to measure the knowledge of HPV infection in an Aboriginal and Torres Strait Islander population. In conclusion, the HPV-KT is a psychometrically robust tool with potential to improve. The addition of items assessing specifications of HPV infection, natural history and behaviour will improve the reliability and usability to assess the level of accurate knowledge about HPV infection. We recommend its application among Aboriginal and Torres Strait Islander communities considering local factors, personal circumstances and socio-economic prerequisites.

## Declaration of Competing Interest

The authors declare that they have no known competing financial interests or personal relationships that could have appeared to influence the work reported in this paper.

## Data Availability

The data that has been used is confidential.
