# Supplementary material for: Development and validation of an HPV infection knowledge assessment scale among Aboriginal and Torres Strait Islander Peoples
Source: Vaccine X. 2023 May 24;14:100317. doi: 10.1016/j.jvacx.2023.100317 (PMC10241973; doi:10.1016/j.jvacx.2023.100317)
Supplement: Supplementary data 1 [file mmc1.docx]

**SUPPLEMENTARY FILES**

**Ten-item network model**

Figure S1: Item stability of the initial HPV-KT network model (10 items)


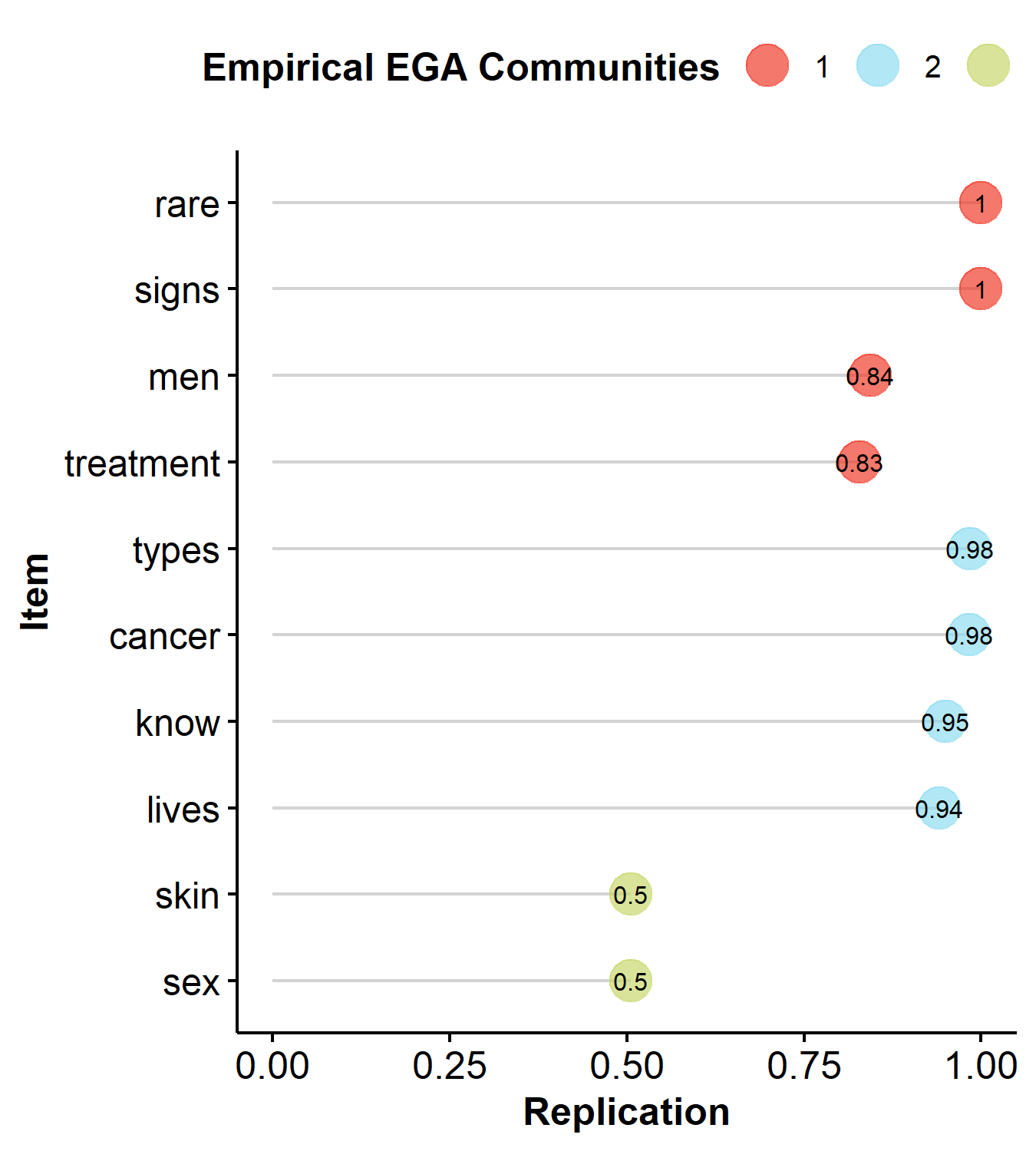


Note. The y-axis indicates the items. The circles are coloured according to their Walktrap-identified community. The x-axis indicates the proportion of times the item clustered with the Walktrap-identified community across the bootstrap samples. The number inside the circle indicates the proportion of times the item clustered with the Walktrap-identified community for each individual item.

Figure S2. Node centrality estimates of the HPV-KT scale (10 items)

**
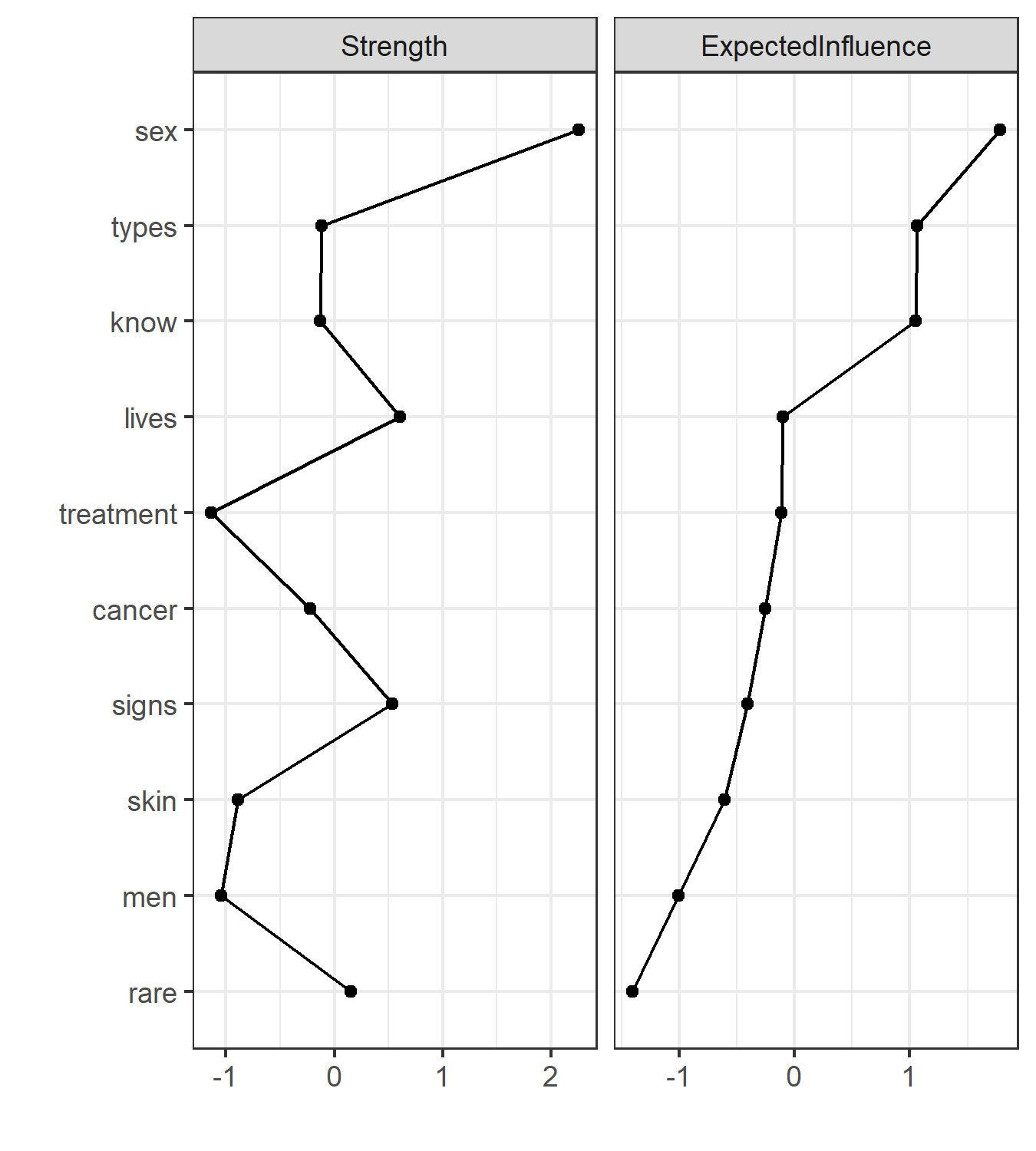
**

**Nine-item network model**

Figure S3: Initial network models of the HPV-KT scale (9 items)

**
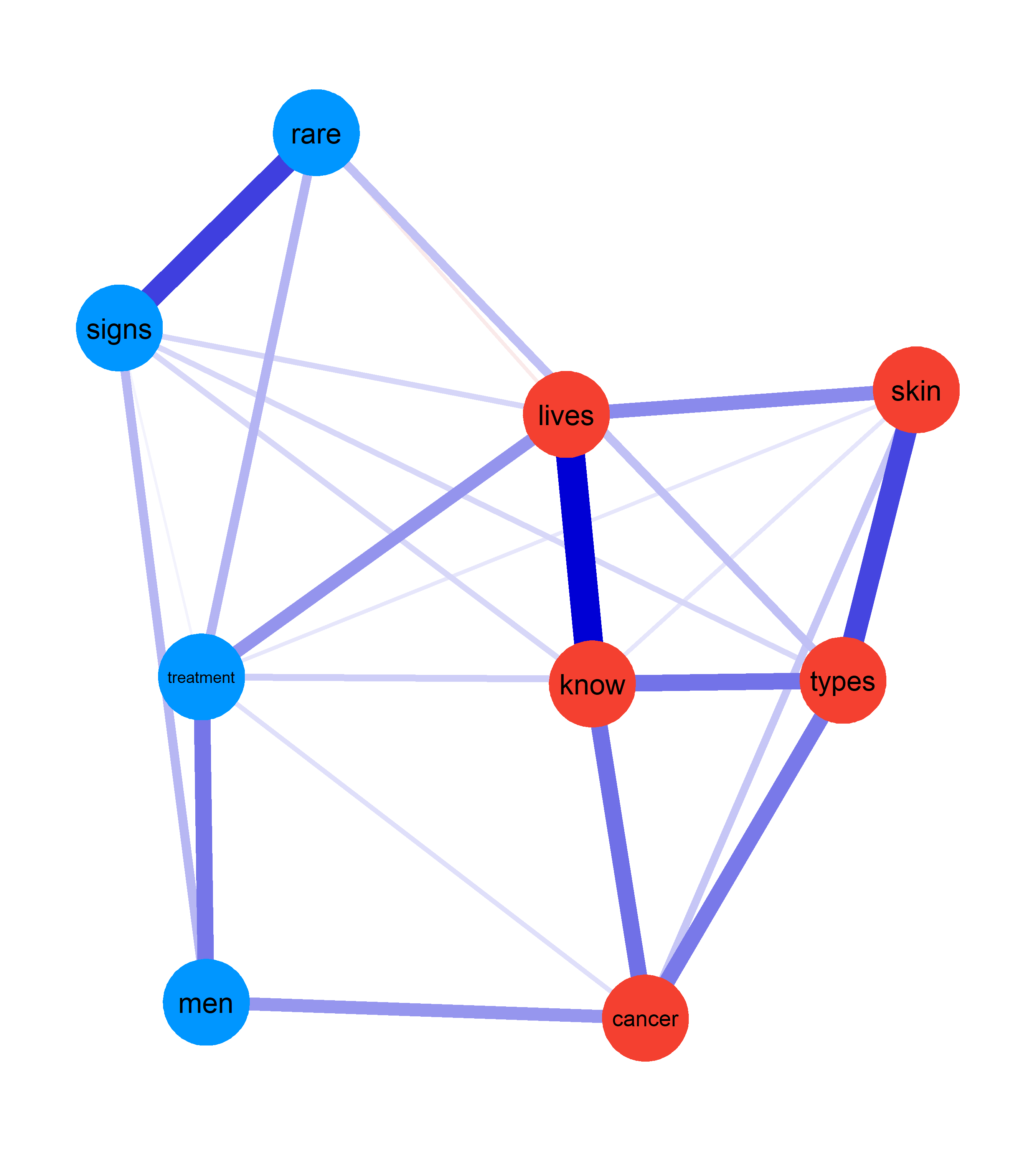
**

Note. Nodes represent items and edges represent partial correlations between items.

Figure S4: Item stability of the initial HPV-KT network model (9 items)

**
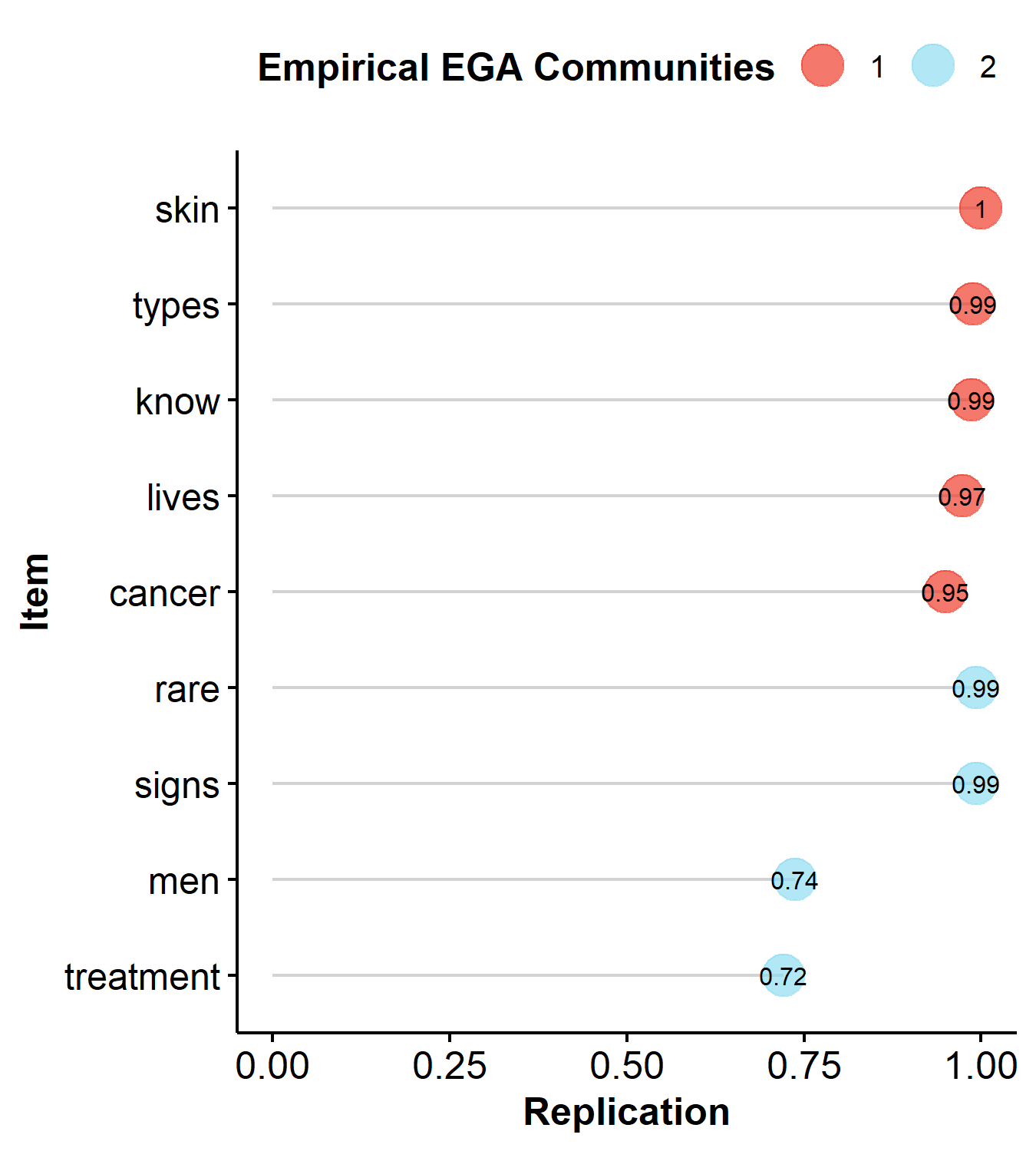
**

Note. The y-axis indicates the items. The circles are coloured according to their Walktrap-identified community. The x-axis indicates the proportion of times the item clustered with the Walktrap-identified community across the bootstrap samples. The number inside the circle indicates the proportion of times the item clustered with the Walktrap-identified community for each individual item.

Figure S5. Node centrality estimates of the HPV-KT scale (9 items)

**
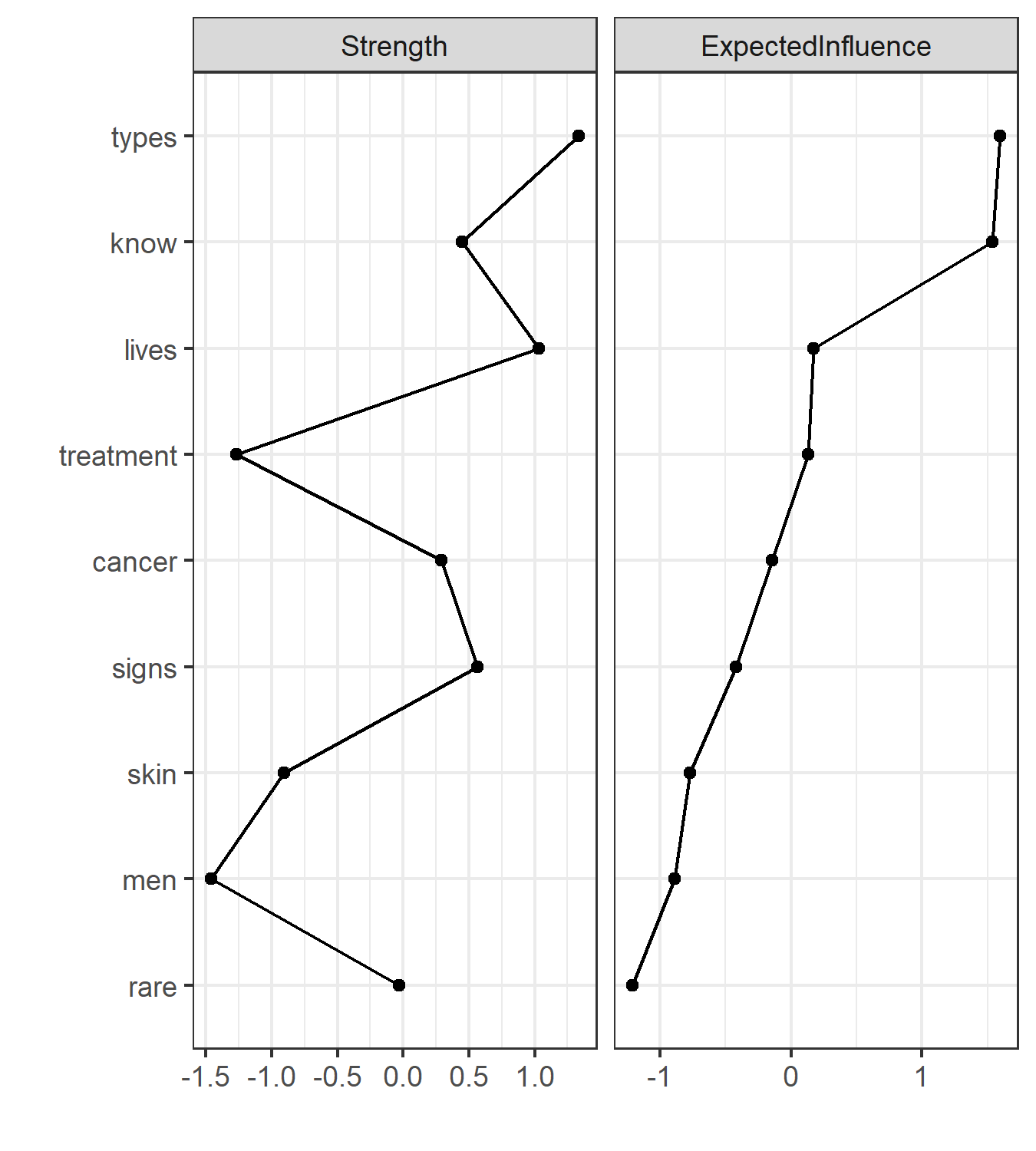
**

**7-item network model**

Table S1. Correlation matrix of the final HPV-KT network model.

|  | rare | signs | skin | types | men | treatment | lives | know | cancer |
| --- | --- | --- | --- | --- | --- | --- | --- | --- | --- |
| rare | 1.000 |  |  |  |  |  |  |  |  |
| signs | 0.486 | 1.000 |  |  |  |  |  |  |  |
| skin | 0.129 | 0.199 | 1.000 |  |  |  |  |  |  |
| types | 0.351 | 0.382 | 0.639 | 1.000 |  |  |  |  |  |
| men | 0.228 | 0.321 | 0.204 | 0.258 | 1.000 |  |  |  |  |
| treatment | 0.331 | 0.321 | 0.386 | 0.437 | 0.434 | 1.000 |  |  |  |
| lives | 0.097 | 0.351 | 0.554 | 0.523 | 0.293 | 0.470 | 1.000 |  |  |
| know | 0.257 | 0.376 | 0.551 | 0.654 | 0.309 | 0.417 | 0.719 | 1.000 |  |
| cancer | 0.200 | 0.168 | 0.506 | 0.614 | 0.408 | 0.408 | 0.482 | 0.618 | 1.000 |

Figure S6. Node centrality estimates of the HPV-KT scale (7 items)

**
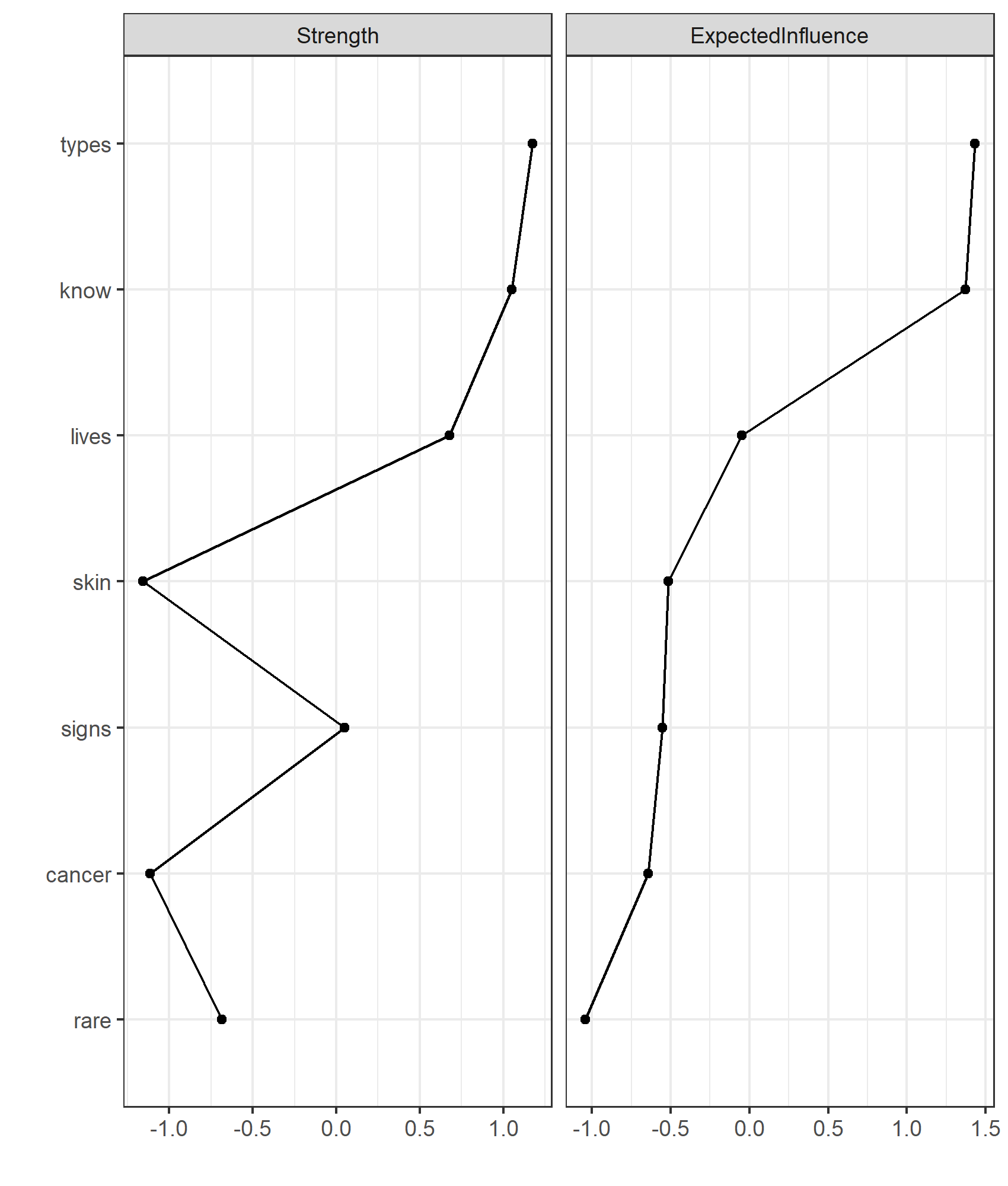
**
